# Draft genome sequencing and assembly of Risso's dolphin, *Grampus griseus*

**DOI:** 10.7150/jgen.78761

**Published:** 2023-01-01

**Authors:** Jayan D.M. Senevirathna, Ryo Yonezawa, Taiki Saka, Yuka Hiramatsu, Ashley Rinka Smith, Yoji Igarashi, Kazutoshi Yoshitake, Shigeharu Kinoshita, Noriko Funasaka, Shuichi Asakawa

**Affiliations:** 1 Laboratory of Aquatic Molecular Biology and Biotechnology, Department of Aquatic Bioscience, Graduate School of Agricultural and Life Sciences, The University of Tokyo, Japan.; 2 Department of Life Sciences and Chemistry, Graduate School of Bioresources, Mie University, Japan.; 3 Department of Life Sciences, Graduate School of Bioresources, Mie University, Japan.; 4 Department of Animal Science, Faculty of Animal Science and Export Agriculture, Uva Wellassa University, Sri Lanka.

**Keywords:** Cetacean, Dolphin, Genome sequencing, Hybrid genome assembly, Vertebrate genomes

## Abstract

The Risso's dolphin (*Grampus griseus*) is one of the migratory marine mammals and they have commonly dispersed in tropical and temperate seas. It is a least concerned species in the IUCN red list of threatened species. However, their population size and factors affecting their population structure are unknown. Due to the wide distribution of this species, their populations might be genetically stable and less structured. To support genetic studies on dolphins and other marine mammals, we assembled the draft genome of Risso's dolphin that was found in Japan. The tissue samples were used to extract high molecular DNA and subjected to sequencing by Illumina HiSeq X, Oxford Nanopore MinION, and Bionano Saphyr. The assembled hybrid genome was 75.9% of complete eukaryotic BUSCOs and the genome size was 2.256 Gb with 2.042 Mb of scaffold N50. *De novo* assembly of this genome by Bionano Saphyr recovered 2.036 Gb total genome map length and structural variations. The gene structures of this draft genome were identified by BRAKER2, and 9947 genes were recovered. The data will be useful for future studies of cetaceans.

## Introduction

Due to several factors, marine mammals are vulnerable, therefore conservation of genetic resources, population genetic analysis, level of inbreeding, and diversity of these animals are currently highly important to study. Risso's dolphin (*Grampus griseus*) is a well-focused migratory dolphin species. They are abundant in all tropical and temperate seas but may not cross polar regions. Risso's dolphin shows remarkable color patterns in different ages, a unique dentition with a smaller number of teeth compared to other cetaceans, a distinctive shape of the head with less/no beak, around three meters long body size of adults, and social animals with less sexual dimorphism 1. The family Delphinidae, oceanic dolphins include toothed whale species of similar/different sizes, colors, and shapes. This group of animals always represents taxonomic difficulties in traditional methods, therefore, interest in genetic studies. However, a recent study reported that *Grampus griseus* belongs to the sub-family Globicephalinae 2 paraphyletic to *Orcaella* spp. 3. The genome sizes of cetaceans' ranges from 2-5 Gb 4, and they show gene mutations when adapting to aquatic life 5. Hence, genome evaluation of cetaceans is essential to identify phylogeny, closest relatives, and convergent evolution.

We sequenced and assembled data from different next-generation sequencing (NGS) tools to produce a standard draft genome of Risso's dolphin. Assembly of sequence reads appropriately is necessary to have a reliable reference genome 6. In this study, we aimed to select recently developed assembly tools that suit large vertebrate genomes with a high background noise. Finally, Bionano Saphyr was intended to produce a hybrid assembly of draft genomes of Risso's dolphin into contig/scaffold level. The data from our Risso's dolphin genome project will be useful for comparative studies of cetaceans in the future.

## Materials and Methods

### Sample preparation and sequencing

Tissue samples of Risso's dolphin were received from Taiji Fisheries Association, Wakayama, Japan (sample ID: 19TK409, male) under the cooperation of biological surveys by the National Research Institute of Far Seas Fisheries, Japan Fisheries Research and Education Agency. A voucher sample (UMUT RV20210330-01) has been preserved at The University Museum, The University of Tokyo, Tokyo, Japan. Initially, a muscle sample was used to extract DNA following the protocol of DNeasy Blood & Tissue Kits (Qiagen, Hilden, Germany). Then, the Nextera DNA Flex Library Prep kit (Illumina, San Diego, CA, USA) was used to prepare the DNA library (insert size of 400 bp) and short-read sequencing was done by Illumina HiSeq X Ten platform (Macrogen, Tokyo, Japan). High molecular weight (HMW) DNA was extracted from a blood sample using the NucleoBond HMW DNA extraction kit (MACHEREY-NAGEL, Düren, Germany). Long-read library was prepared by a ligation sequencing kit and real-time sequencing was performed by the Oxford Nanopore MinION system (Oxford Nanopore Technologies, Oxford, UK) in our laboratory. A liver sample was used for DNA extraction and sequencing was done by Bionano Saphyr (BioNano Genomics, San Diego, CA, USA).

### Draft genome assembly

Initially, short-read sequencing data was assembled with long-read data using the Long Reads Scaffolder (LRScaf 1.8.1, https://github.com/shingocat/lrscaf) 7. The suitable statistics for this algorithm were checked for 1,000 times array by grid engine technique. The best assembly was performed with 1,000 bp minimum contig length and 1,300 bp maximum overhang length. At first, short reads were aligned against the draft assemblies by minimap2 and improved via the LRScaf using long read scaffolds. The Bionano Saphyr system (ASONE, Japan) generated a *de novo* genome (Tools Version 1.6.1, Bionano Genomics, San Diego, CA, USA) and further aligned by scaffolding with Bionano optical mapping to fill the gaps in the hybrid genome that generated by the LRScaf scaffolder. Genome statistics were produced by the Assembly-stats (https://github.com/sanger-pathogens/assembly-stats) and the ABySS 8 (https://github.com/bcgsc/abyss).

### Repeat analysis and gene prediction

Finally, the hybrid genome of Risso's dolphin that prepared by Bionano + MinION + HiSeq X technologies was used for gene prediction. The completeness of the hybrid draft genome of Risso's dolphin was checked by BUSCO analysis 9,10 against Eukaryotic BUSCOs (version 3.0.2). Additionally, we used AUGUSTUS 11 and BRAKER2 12 for genome annotation and gene prediction with RNA-seq data (DDBJ Bioproject - PRJDB11720). The predicted transcript list was used to identify potential genes via Enrichr 13. This gene set was used to identify functional annotations and gene ontologies via the WEB-based GEne SeT AnaLysis Toolkit 14.

## Results and Discussion

The predicted genome size of Risso's dolphin is about 2.3-2.7 Gb comparable to other species in the family Delphinidae, like *Tursiops truncates* (PRJNA625792), *Sousa chinensis* (PRJNA449414), *Platanista minor* (PRJNA399467), and *Lipotes vexillifer* (PRJNA232751, PRJNA174066). We sequenced both short and long-read libraries and generated around 1,875.3 Gb of DNA data. According to Table 1, we received 987,466,434 short-reads from HiSeq X and 1,978,053 of long-reads from MinION. The first hybrid assembly of HiSeq X + MinION produced 473,906 contigs. Finally, the second assembly of HiSeq X + MinION + Bionano produced 470,000 bp of effective contigs (Table 2). Of these, 822 Mb (36%) out of 2.256 Gb was able to scaffold, and 1.01 Mb was the only Scaffold N50 that could be stretched out from Bionano. As an advantage of the hybrid approach of HiSeq X short-reads + MinION long-reads + Bionano scaffolding, our assembly method of Risso's dolphin genome has been improved and completed 75.9% of eukaryotic BUSCOs (Table 3).

**Figure 1:**
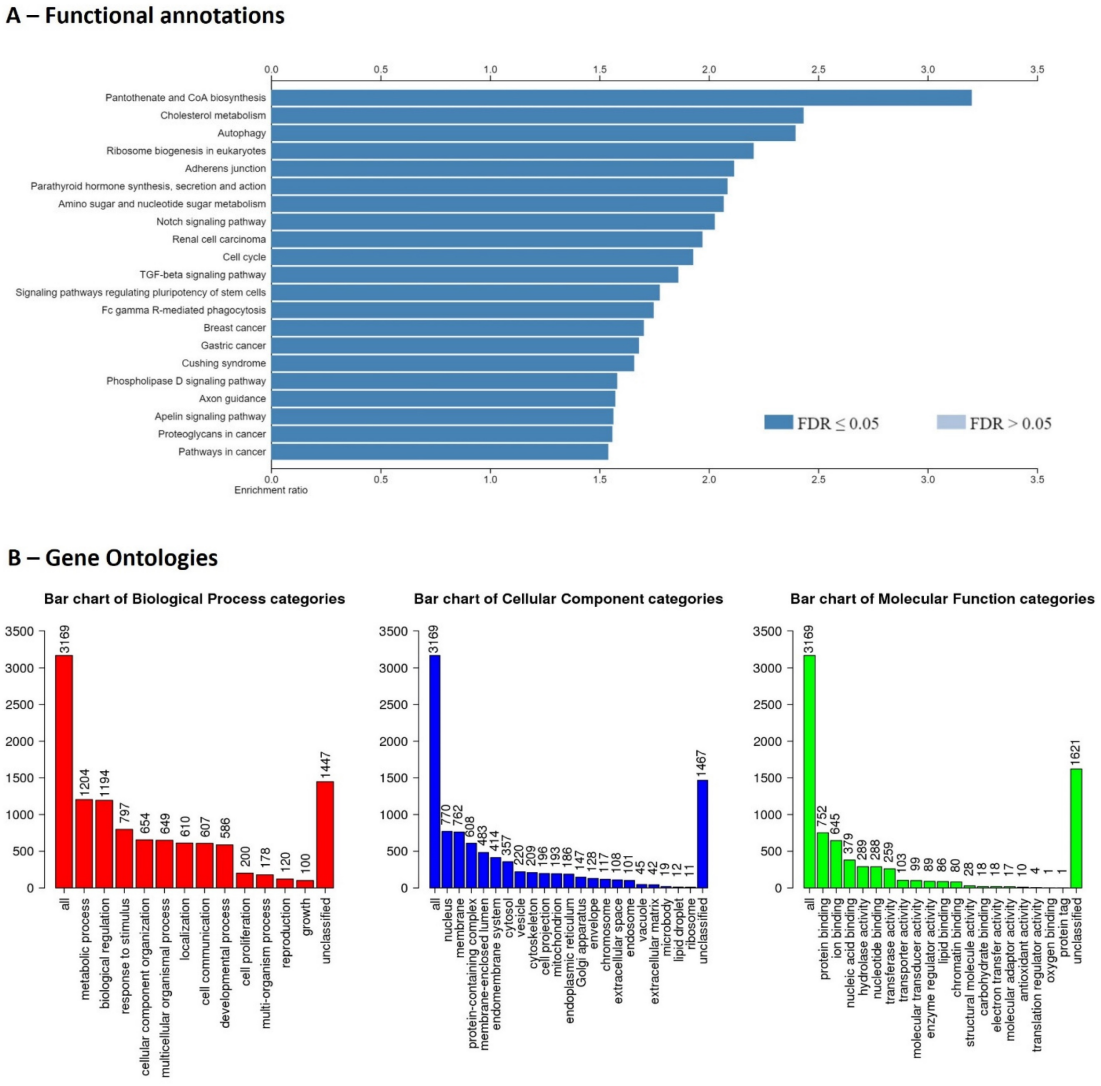
Functional enrichments (A) and gene ontologies (B) for recovered genes of the draft genome of Risso's dolphin.

Although the genome completion is 75.9%, the Bionano genomics has identified structural variations. In summary, 74 deletions, 232 insertions, and 2,421 inter-chromosomal translocation breakpoints were identified. The gene structure information file (gene transfer format) produced by the BRAKER2 pipeline showed 494,731 coding sequences (exons), 349,409 introns, 145,716 start codons, and 136,520 stop codons, and 175,481 transcripts. The predicted transcripts have been identified by NCBI NT top hit (>70%) and UniProt Swiss-Prot top hit. There were 31 transcripts with ≥ 10,000 bp length, ≥ 95% NCBI NT similarity for 15 proteins (MUC5B, OBSCN, WDR90, RYR1, UBR4, BSN, CELR3, PCD16, ZN469, RN213, SZT2, KMT2D, LRP1, HMCN2, PLEC). Finally, these transcripts were filtered by Enrichr to identify potential protein coding gene IDs and it was 9,947 genes. In which, 3,169 gene IDs were unambiguously mapped to 3,169 unique Entrez gene IDs and 6,778 gene IDs could not be mapped to any Entrez gene ID. Out of the identified 3,169 genes, pantothenate and CoA biosynthesis, and cholesterol metabolism were highly enriched functions (Figure 1). Identification of lipid metabolism-related genes will be important for studies of convergent evolution and macroevolutionary characteristics of cetaceans 15.

## Figures and Tables

**Table 1:** Raw data and assembly statistics

Statistics	HiSeq X raw data	MinION raw data	*De novo* Bionano	Hybrid assembly I (MinION + HiSeq X)	Hybrid assembly II (Bionano + MinION + HiSeq X)
Total read length (bp)	11,790,000,000	7,738,000,000	671,653,189,000	2,575,341,302	2,256,000,000
Number of reads/contigs	987,466,434	1,978,053	2,491,307	473,906	470,000
Sequencing depth	56.47X	6.4X	46.17X	-	-
Average read length (bp)	4,174.53	4,000.93	-	5,434.29	6,384.70
Longest read length (bp)	192,510	192,510	-	766,882	27,508,099
Maximum contig length (bp)	192,509	192,509	-	719,756	13,170,000
Minimum contig length (bp)	500	500	-	500	500
N 20 (bp)	22,270	26,608		154,740	838,827
N50 (bp)	10,435	11,605	902,000	78,917	80,163
Counts @ N50	316,595	184,732	-	9,557	2,680
N100 (bp)	52	52	-	200	2
Counts @ N100	2,878,053	1,978,053	-	473,906	470,000
N20 (bp)	22,270	26,608	-	142,933	838,827
L50	306,128	177,054	-	8,989	4,290

**Table 2:** Bionano statistics

Statistics	Original BNG	Original NGS	NGS used in Hybrid scaffold	Hybrid Scaffold	Hybrid + not scaffolded NGS
Count	4,668	473,906	7,627	1,580	470,000
Mean length (Mb)	0.436	0.005	0.118	0.837	0.006
N50 (Mb)	0.902	0.079	0.128	2.042	0.116
Max length (Mb)	20.527	0.767	0.767	27.508	27.508
Total Length (Mb)	2,036.348	2,575.341	896.478	1,321.948	3,000.811

**Table 3:** BUSCOs of draft genomes

BUSCOs	Illumina HiSeq X	Hybrid HiSeq X + MinION	Hybrid HiSeq X + MinION + Bionano
Complete	57.1%	78.2%	75.9%
Single-copy	54.1%	72.6%	70.0%
Duplicated	3.05	5.6%	5.9%
Fragmented	18.8%	9.6%	10.6%
Missing	24.1%	12.2%	13.5%
Total BUSCO groups	303		
